# FA1 Induces Pro-Inflammatory and Anti-Adipogenic Pathways/Markers in Human Myotubes Established from Lean, Obese, and Type 2 Diabetic Subjects but Not Insulin Resistance

**DOI:** 10.3389/fendo.2013.00045

**Published:** 2013-04-05

**Authors:** Basem M. Abdallah, Henning Beck-Nielsen, Michael Gaster

**Affiliations:** ^1^Molecular Endocrinology Laboratory (KMEB), Odense University Hospital, University of Southern DenmarkOdense, Denmark; ^2^Department of Endocrinology, Odense University Hospital, University of Southern DenmarkOdense, Denmark; ^3^Laboratory of Molecular Physiology, Department of Pathology, Odense University Hospital, University of Southern DenmarkOdense, Denmark

**Keywords:** Dlk1, FA1, Pref-1, insulin resistance, human myotubes, obesity, skeletal muscle, type 2 diabetes

## Abstract

**Aims:** Delta like 1/fetal antigen 1 (Dlk1/FA1) is a protein secreted by hormone producing cells in adult human and mice that is known to inhibit adipogenesis. Recent studies demonstrated the role of Dlk1/FA1 in inducing insulin resistance in mice. To investigate the involvement of circulating Dlk1/FA1 in insulin resistance and type 2 diabetes in human subjects, we studied the effects of chronic FA1 on the intermediary metabolism in myotubes established from lean, obese, and type 2 diabetic (T2D) subjects.

**Methods:** Myotube cultures were established from lean and obese control subjects, and obese T2D subjects and treated with soluble FA1 for 4 days supplemented with/without palmitate (PA). Lipid- and glucose metabolism were studied with labeled precursors while quantitative expression of genes was analyzed using real-time PCR.

**Results:** Diabetic myotubes express significantly reduced insulin stimulated glucose metabolism compared to lean myotubes and a significantly decreased basal PA oxidation. Chronic FA1 exposure did not affect the intermediary metabolism in myotubes. Insulin sensitivity of glucose and lipid metabolism was not affected by chronic FA1 exposure in myotubes established from lean, obese, and T2D subjects. Instead, chronic FA1 exposure induced pro-inflammatory cytokines expression (IL-6 and CCL2) in association with reducing adipogenic markers (ADD1, AP2, CD36, and PPARg2) in myotubes. Consistent with this observation, addition of FA1 to cultured myotubes was show to significantly inhibit their differentiation into adipocyte.

**Conclusion:** Our results exclude direct effects of FA1 on glucose and lipid metabolism in cultured myotubes established from lean, obese, and T2D subjects. Therefore, the pathogenesis of FA1-induced IR might mainly be mediated via the FA1-induced stimulation of pro-inflammatory cytokines, which on turn inhibit adipogenesis in human myotubes.

## Introduction

Delta like 1/Pre-adipocyte factor-1 (Dlk1/Pref-1) is a transmembrane protein that contains six cysteine-rich EGF-like repeats in its extracellular domain, similar to those found in the Delta/Notch/Serrate family of signaling molecules (Laborda et al., 1993; Fleming, 1998). Dlk1 is a broadly expressed protein by most embryonic and fetal tissues in human and mice and its expression is dramatically down-regulated to become localized only to the hormone secreting cells in the pituitary gland, pancreatic islets, adrenal glands, and testis (Floridon et al., 2000; Yevtodiyenko and Schmidt, 2006). Dlk1 plays a critical role in modulating cell fate decisions throughout development (Laborda, 2000) and has been identified as a negative regulator of adipocyte differentiation (Smas and Sul, 1993). Mice deficient in *Dlk1* displayed growth retardation, obesity, skeletal malformations, and abnormalities of hematopoiesis (Moon et al., 2002; Sakajiri et al., 2005). Also, in the human syndrome of maternal uniparental disomy (UPD)14 (where *Dlk1* is silent), patients exhibit obesity, hypotonia, premature puberty, macrocephaly, short stature, and small hands (Berends et al., 1999).

The extracellular domain of the Dlk1 is proteolitically cleaved by tumor necrosis factor alpha converting enzyme (TACE) (Wang and Sul, 2006) and shed into the circulation as a soluble active form named fetal antigen 1 (FA1) (Jensen et al., 1994). Therefore, Dlk1/FA1 can function in a paracrine/endocrine fashion (Jensen et al., 1993). Circulating Dlk1/FA1 is present at very high levels in serum/amniotic fluid of pregnant human and mice (Bachmann et al., 1996; Jensen et al., 1997). In addition, the serum level of FA1 is elevated in some human pathological conditions including neurofibromatosis (Jensen et al., 1999), renal failure (10 times higher) (Jensen et al., 1997), and small cell lung cancer patients (10–1000 times higher) (Harken et al., 1999).

Consistent with to its role in adipogenesis, we have shown that systemic administration of FA1 in adult mice led to reduce both fat and bone mass in a dose-dependent manner (Abdallah et al., 2007b).

Recently, transgenic mice with high circulating level of FA1 were shown to display a lipodystrophic phenotype in association with increased whole body insulin resistance suggesting an endocrine function for FA1 in insulin resistance and T2D (Villena et al., 2008).

In our attempt to investigate the potential role of FA1 in insulin sensitivity and T2D of human subjects, we studied the chronic effect of dlk1/FA1 on insulin sensitivity, and lipid metabolism in myotube cultures established from lean, obese, and type 2 diabetic (T2D) (Gaster et al., 2002) subjects. Our detailed biochemical and molecular analysis revealed an immune-modulatory effect of FA1 on increasing the production of pro-inflammatory cytokines in cultured myotubes without affecting their insulin sensitivity.

## Materials and Methods

### Materials

Dulbecco’s modified Eagle’s medium, fetal calf serum (FCS), penicillin-streptomycin-amphotericin B, and trypsin-EDTA were obtained from Invitrogen (Invitrogen, Scotland, UK). Ultroser G was purchased from Pall Biosepra (Cergy-Saint-Christophe, France). Protein assay kit was purchased from Bio-Rad (Copenhagen, Denmark). Palmitic acid, l-carnitine, and ECM-gel were purchased from Sigma Chemical Co. (St. Louis, MO, USA). Bovine serum albumin (BSA; essentially fatty acid free) was from Calbiochem (VWR, Roskilde, Denmark). Insulin Actrapid was from Novo Nordisk (Bagsvaerd, Denmark).

### Fetal antigen 1

The full soluble ectodomain, active form of Dlk1 protein named FA1, was purified by immuno-specific affinity chromatography from human amniotic fluid and kindly provided by Dr. Bøger Teisner (Immunology Dept., OUH, Denmark) as described previously (Jensen et al., 1993).

### Human study subjects

Eight lean, eight obese control subjects, and eight obese T2D patients participated in the study (Table 1), and their clinical characteristics have previously been published (Ortenblad et al., 2005; Gaster and Beck-Nielsen, 2006). All subjects gave written, informed consent, and the study was approved by the local ethics committee of Funen and Vejle County. Muscle biopsies were obtained from the vastus lateralis muscle by needle biopsy under local anesthesia. Diabetic patients were treated with either diet alone or in combination with sulfonylurea, metformin, or insulin withdrawn 1 week before the study. The patients suffered from no diabetic complications except for simplex retinopathy. The control subjects had normal glucose tolerance and no family history of diabetes.

**Table 1:** **Clinical characteristics of the study subject**.

	Control, lean	Control, obese	T2D
*n*	8	8	8
Age (years)	51 ± 1	49 ± 1	50 ± 2
Weight (kg)	71.5 ± 3.7	109.3 ± 7.6*	106.6 ± 3.6*
BMI (kg/m^2^)	24.3 ± 0.6	34.4 ± 1.9*	34.5 ± 1.0*
Fasting plasma glucose (mM)	5.7 ± 0.1	5.9 ± 0.1	9.6 ± 0.7^#^
Fasting serum insulin (pM)	26.6 ± 6.8	53.6 ± 5.9*	102.5 ± 10.4^#^
Glucose infusion rate (mg/min)	375.3 ± 20.4	232.9 ± 21.9.3*	124.3 ± 17.8^#^
HbA_1c_ (%)	5.6 ± 0.1	5.5 ± 0.1	7.6 ± 0.6^#^
Fasting total cholesterol (mM)	5.25 ± 0.28	5.48 ± 0.57	5.31 ± 0.39
Fasting LDL cholesterol (mM)	2.90 ± 0.26	3.39 ± 0.45	3.18 ± 0.33
Fasting HDL cholesterol (mM)	1.82 ± 0.17	1.44 ± 0.20	1.36 ± 0.03*
Fasting plasma triglyceride (mM)	1.19 ± 0.19	1.41 ± 0.21	1.73 ± 0.31

Data are means ± SE. *Significant different from the lean controls (*P* < 0.05), ^#^significant different from the lean and obese controls (*P* < 0.05).

### Cell culture

Cell cultures were established as previously described (Gaster et al., 2001a,b,c). In brief, muscle tissue was minced, washed, and dissociated for 60 min in 0.05% trypsin-EDTA. The cells obtained were seeded for up-scaling on ECM-gel coated dishes after 30 min of pre-plating. Growth medium contains DMEM supplemented with 2% FCS, 2% Ultroser G, 50 U/ml penicillin, 50 μg/ml streptomycin, and 1.25 μg/ml amphotericin B. Cells were sub-cultured twice before final seeding. At 75% confluence, the growth medium was replaced by basal medium (DMEM supplemented with 2% FCS, 50 U/ml penicillin, 50 μg/ml streptomycin, 1.25 μg/ml amphotericin B, and 25 pmol/l insulin) in order to induce differentiation. The cells were cultured in humidified 5% 2CO_2_ atmosphere at 37°C, and medium was changed every 2–3 days.

### Experimental design

Human myotubes established from lean, obese, and T2D subjects were allowed to differentiate under physiological conditions of insulin (25 pmol/l) and glucose (5.5 mmol/l). All myotube cultures were used for analysis day eight after onset of differentiation. Myotubes were exposed to three different protocols: (1) the last 4 days myotubes established from all three groups were exposed with/without 3.0 μg/ml FA1 followed by subsequent determination of glucose uptake, oxidation and storage, lipid uptake, and oxidation at baseline and during acute insulin stimulation and protein content. RNA were isolated from sister cultures. (2) The last 4 days myotubes established from lean subjects were exposed with/without 3.0 μg/ml FA1 supplemented with 0.4 mmol/l PA followed by subsequent determination of glucose uptake, oxidation and storage, lipid uptake, and oxidation at baseline and during acute insulin stimulation and protein content. RNA were isolated from sister cultures. (3) lean myotubes were exposed for 0.4 mmol/l PA with/without 3.0 μg/ml FA1 day 4 and PA incorporation into the myotubes at various time points determined by scintillation proximity assay (SPA) method (Gaster, 2009b) for 7 days (168 h). PA: BSA molar ratio was 2.5:1.

### Substrate oxidation

Glucose and palmitate (PA) oxidation was determined by a 96 multi-well tracer technique as previous described (Gaster, 2009a). Substrate oxidation was monitored by incubating myotubes with [1-14C]-PA (2.0 μCi/ml) in a final concentration of 0.4 mmol/l PA and [14C(U)]-glucose (2.0 μCi/ml) in a final concentration of 5.0 mmol/l glucose with subsequent capture of liberated 14CO_2_ for 4 h at 37°. Trapped radioactivity was determined on a Microbeta counter (PerkinElmer, Finland).

### Adipocyte differentiation

AIM Cells were seeded at 3 × 10^4^ cells/cm^2^ in 60 cm^2^ petri-dishes (for RNA isolation) or in six-well plates (for histochemical staining and flow cytometry studies) and cultured in a standard growth medium. At 90–100% cell confluence, the medium was replaced by high-glucose Dulbecco’s modified MEM (DMEM; Gibco Invitrogen) containing 10% FCS and supplemented with adipogenic-induction mixture (AIM) [containing 10^−7^ M dexamethasone (dex), 0.45 mM isobutyl methyl xanthine (IBMX), 2 × 10^−6^ M insulin (all from, Sigma-Aldrich, Vallensbaek strand, Denmark), and 1 μM Rosiglitazone [(BRL49653; Novo Nordisk, Bagsvaerd, Denmark)]. The adipogenic medium was replaced every 3 days. Cells differentiated into adipocytes were washed twice in PBS and lipid staining was performed using Oil Red O stain.

### Glucose and lipid uptake

Glucose uptake was measured by capturing 2-[1-14C]-deoxy-glucose and lipid uptake was measured as the incorporation of [1-14C]-PA (2.0 μCi/ml) as previously described (Gaster and Beck-Nielsen, 2004, 2006). Radioactivity was determined on a Microbeta counter (PerkinElmer, Finland).

### Glycogen synthesis

Glycogen synthesis was measured as previous described (Gaster and Beck-Nielsen, 2004) in 96 well plates. Radioactivity was measured with a Microbeta counter (PerkinElmer, Finland).

### PA incorporation in intracellular lipids

Incorporation of PA over time into human myotubes was estimated by an SPA (Gaster, 2009b) taking the advantage of the fact that the accumulated radioactivity provides signal stronger than the signal coming from the media. Differentiated myotubes were exposed to increasing C-14 labeled PA concentrations (0.4 mmol/l) with/without 3.0 μg/ml FA1 for 7 days. PA incorporated into the myotubes was monitored regularly by measuring accumulation of radioactivity (Microbeta, PerkinElmer, Finland).

### RNA isolation and real-time

Total RNA was isolated from human myotubes using a single step method with TRIzol (Invitrogen A/S, Tastrup, Denmark) according to the manufacturer’s instructions. The integrity and purity of total RNA was verified spectrophotometrically and by gel-electrophoresis on 0.8% SeaKem agarose (BMA, Hellerup, Denmark). cDNA was synthesized from 5 μg of total RNA using a commercial revertAid H minus first strand cDNA synthesis kit (Fermentas, Copenhagen, Denmark) according to manual instructions. Real-time PCR (RT)-PCR was performed in iCycler IQ detection system (Bio-Rad, Herlev, Denmark) by using SYBR^®^  Green I as a double-strand DNA-specific binding dye. Thermocycling was performed in a final volume of 20 μl containing 3 μl of cDNA sample (diluted 1:20), 20 pmole of each primer, and 2× iQ™  SYBR^®^  Green Supermix (Bio-Rad). The quantification of each target gene and β-actin mRNA using primers in Table 2 was performed in separate tubes. Gene expression levels for each target gene were calculated using the comparative Ct method [(1/(2ΔCt) formula, where ΔCt is the difference between Ct target and Ct-reference] after normalization to β-actin mRNA (PerkinElmer’s User Bulletin No. 2). Data were analyzed using optical system software version 3.1 (Bio-Rad) and Microsoft Excel 2000 to generate relative expression values (Frederiksen et al., 2008).

**Table 2:** **Primer sequences used for real-time PCR gene expression analysis**.

Gene	Primer sequence		Product size (bp)
**REFERENCE GENES**			
β-Actin	5′-TGTGCCCATCTACGAGGGGTATGC-3′	F	433
	5′-GGTACATGGTGGTGCCGCCAGACA-3′	R	
**CYTOKINE GENES**			
Dlk1	5′-CTGGACGGTGGCCTCTATGAATG-3′	F	130
	5′-ATCATCCACGCAGGTGCCTC-3′	R	
IL-1b	5′-AGGAAGATGCTGGTTCCCTGC-3′	F	126
	5′-CAGTTCAGTGATCGTACAGGTGC-3′	R	
IL-6	5′-CCACACAGACAGCCACTCACCTC-3′	F	276
	5′-CTGGCTTGTTCCTCACTACTCTC-3′	R	
CC3	5′-CCTGCTACTAACCCACCTCC-3′	F	139
	5′-AACAGTGACTGGAACATCCCC-3′	R	
CD36	5′-AGTCACTGCGACATGATTAATGGT-3′	F	74
	5′-CTGCAATACCTGGCTTTTCTCAA-3′	R	
GLUT1	5′-GGCCAAGAGTGTGCTAAAGAA-3′	F	201
	5′-ACAGCGTTGATGCCAGACAG-3′	R	
GLUT4	5′-TGGGCGGCATGATTTCCTC-3′	F	88
	5′-GCCAGGACATTGTTGACCAG-3′	R	
TNFa	5′-TTCTCGAACCCCGAGTGACAAG-3′	F	375
	5′-CCCTTCTCCAGCTGGAAGACC-3′	R	
CCL2/MCP-1	5′-CCAATTCTCAAACTGAAGCTCGCAC-3′	F	372
	5′-GTTAGCTGCAGATTCTTGGGTTGTG-3′	R	
CPT-1a	5′-TGCTTTACAGGCGCAAACTG-3′	F	338
	5′-TGGAATCGTGGATCCCAAA-3′	R	
LXRa	5′-GAGGGCTGCAAGGGATTCTT-3′	F	330
	5′-GTTACACTGTTGCTGGGCAG-3′	R	
LXRb	5′-GGCGAGGGTGTCCAGCTAA-3′	F	90
	5′-CGGAGAAGGAGCGTTTGTTG-3′	R	
**ADIPOCYTE MARKERS**			
FASN	5′-CTCCGAAGGGCACGAACAC-3′	FR	290
	5′-TAGAGGGAGCCAGAGAGACG-3′		
APM1	5′-TGTTGCTGGGAGCTGTTCTACTG-3′	FR	234
	5′-ATGTCTCCCTTAGGACCAATAAG-3′		
aP2	5′-GCCAGGAATTTGACGAAG TC-3′	FR	220
	5′-TGGTTGATTTTCCATCCC AT-3′		
ADD1	5′-GGAGCCATGGATTGCACTTTC-3′	FR	261
	5′-ATCTTCAATGGAGTGGGTGCAG-3′		

### Statistical analysis

The data in the text, tables, and figures are presented as mean ± SE. The statistical analyses were performed with INSTAT 2.01 (GraphPad, USA). *P* ≤ 0.05 was considered to be significant.

## Results

### Chronic FA1 exposure did not affect the intermediary metabolism of lean myotubes

Increasing serum levels of FA1 was shown to be associated with impairment of insulin sensitivity (Villena et al., 2008). In order to investigate the direct involvement of FA1 in stimulating insulin resistance in myotubes, we studied the effect of soluble FA1 on different parameters of insulin sensitivity in cultured myotubes established from lean subjects. As shown in Table 3, lean myotubes exposed to chronic (4 days; 3.0 μg/ml) FA1 did not express significantly different rates of neither glucose and lipid uptake nor glucose oxidation and glycogen synthesis rates as compared to non-treated control cultures (Table 3).

**Table 3:** **Metabolic characteristics of lean myotubes (*n* = 8) with/without exposure to FA1 (3 μg/ml) for 4 days**.

Metabolic characteristics	−FA1	+FA1 (3 μg/ml)
**GLUCOSE TRANSPORT (nmol/min/mg)**		
Baseline	0.715 (±0.055)	0.680 (±0.062)
Acute insulin stimulation	0.873 (±0.087)*	0.981 (±0.132)*
Insulin effect (ratio)	1.205 (±0.055)	1.424 (±0.099)
**GLYCOGEN SYNTHESIS (nmol/min/mg)**		
Baseline	0.213 (±0.069)	0.217 (±0.035)
Acute insulin stimulation	0.390 (±0.040)*	0.380 (±0.054)*
Insulin effect (ratio)	1.856 (±0.120)	1.811 (±0.131)
**GLUCOSE OXIDATION (nmol/min/mg)**		
Baseline	0.106 (±0.011)	0.099 (±0.014)
Acute insulin stimulation	0.128 (±0.012)*	0.137 (±0.015)*
Insulin effect (ratio)	1.221 (±0.083)	1.370 (±0.201)
**LIPID UPTAKE (nmol/min/mg)**		
Baseline	1.490 (±0.078)	1.420 (±0.089)
Acute insulin stimulation	2.040 (±0.090)**	1.940 (±0.099)**
Insulin effect (ratio)	1.358 (±0.045)	1.354 (±0.050)
**LIPID OXIDATION (pmol/min/mg)**		
Baseline	39.94 (±2.52)	36.66 (±2.78)
Acute insulin stimulation	40.14 (±2.77)	36.83 (±2.77)
Insulin effect (ratio)	1.000 (±0.03)	0.975 (±0.04)

*Data represents mean ± SEM. *P* < 0.05 is considered significant. Baseline conditions refer to an insulin concentration of 25 pmol/l and acute insulin stimulation refers to an insulin concentration of 1 μmol/l*.

*P < 0.05, baseline vs. acute insulin stimulation.

**P < 0.001, baseline vs. acute insulin stimulation.

### Chronic FA1 treatment does not reduce the Insulin sensitivity of the glucose and lipid metabolisms in myotubes established from lean, obese, and T2D subjects

**Figure 1:**
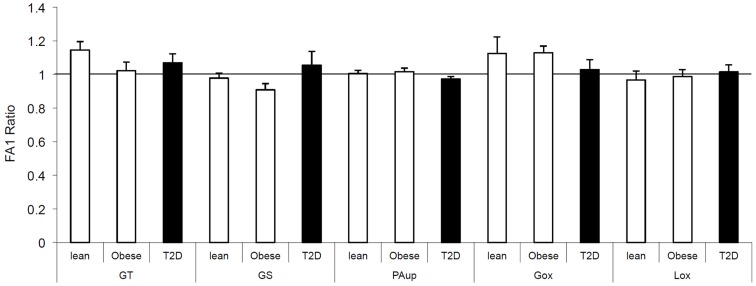
**Effect of FA1 treatment on the insulin sensitivity of cultured myotubes from lean, obese, and T2D subjects**. Human myotubes established from lean, obese, and T2D subjects were differentiated for 4 days under physiological conditions and subsequently treated without (control) or with 3.0 μg/ml FA1 for further 4 days, as described in Section “Materials and Methods.” Glucose uptake (GT), glucose oxidation (Gox), glycogen synthesis (GS), lipid uptake (PAup), and lipid oxidation (Lox) were determined at baseline and during acute insulin stimulation. Data represented as ratio of (insulin stimulation/basal) with/without FA1. Data are shown as mean ± SEM. *N* = 8 in each group.

We also investigated the direct effect of FA1 on insulin and lipid metabolisms in both obese and T2D myotubes. As shown in Figure 1, treatment of obese and diabetic cultured myotubes for 4 days with purified hFA1 did not significantly reduce any of the insulin sensitivity parameters including glucose uptake and oxidation, glycogen synthesis, PA uptake, and oxidation as compared to non-treated control cultures (Figure 1).

### Chronic FA1 exposure induces pro-inflammatory gene expression in human myotubes and inhibits adipogenic markers

**Figure 2:**
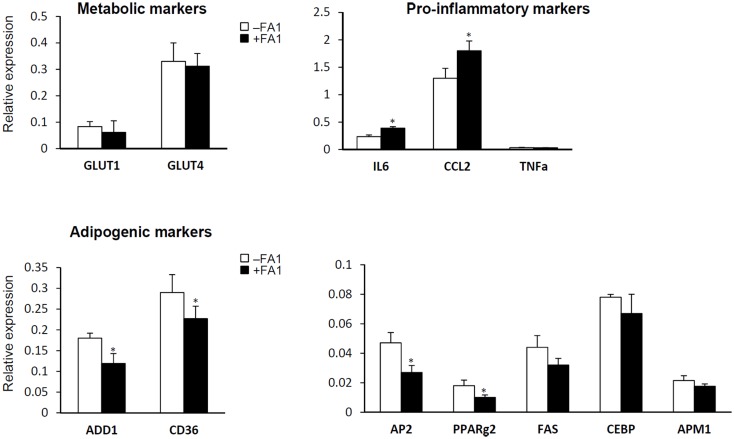
**Effect of FA1 treatment on gene expression of metabolic, pro-inflammatory, and adipogenic markers in cultured myotubes from lean, obese, and T2D subjects**. Cells were differentiated for 4 days under physiological conditions and subsequently treated without (control) or with 3.0 μg/ml FA1 for further 4 days, as described in Section “Materials and Methods.” Gene expression analysis was measured by real-time PCR and the expression analysis of each target gene was represented as relative expression to β-actin. Data from the three groups were pooled as there were no differences between their expression levels of several genes important for various aspects of the intermediary metabolism. Data represented as mean ± SEM (*N* = 8 per each group); *P < 0.05.

We further studied the effect of FA1 treatment on gene expression profile of inflammatory, adipogenic, and glucose transport genes in cultured myotube established from lean, obese, and T2D subjects (Figure 2). As shown in Figure 2, FA1 treatment for 4 days significantly reduce the mRNA expression of adipogenic differentiation markers *ADD1*, *AP2*, *CD36*, and *PPARΥ2* in association with increasing the expression of the pro-inflammatory markers *IL-6* and *CCL2* (Figure 2). Consistent with our biochemical assays, FA1 treatment did not change the mRNA expression of *GLUT1* and *GLUT4* that are important for basal and insulin stimulated glucose uptake respectively.

### FA1 inhibits adipocyte differentiation of human myotubes

**Figure 3:**
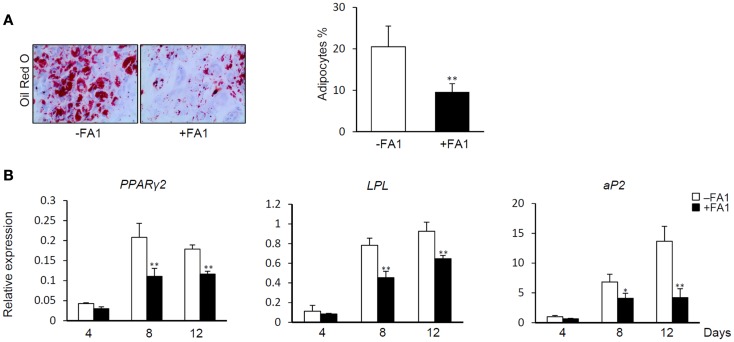
**Effect of FA1 on adipocyte differentiation of myotubes**. **(A)** Soluble active form of Dlk1 (FA1) inhibits the adipocyte differentiation of myotubes. Cultured myotubes established from lean subjects were induced to differentiate into adipocyte without (control) or with 3 μg/ml of purified FA1. Adipocytes were stained at day 12 with Oil red O and quantified by microscopic investigation. **(B)** Expression of adipogenic markers during the time course of differentiation in the absence and the presence of FA1. Gene expression analysis was quantified by real-time PCR and the expression of each target gene was represented as relative expression to β-actin. Data are shown as mean ± SEM of at least three independent experiments; *P < 0.05 and **P < 0.005.

Delta like 1/FA1 has been established as an inhibitor of adipocyte differentiation and fat mass *in vitro* and *in vivo* (Abdallah et al., 2004, 2011b). Thus, we studied the inhibitory effect of FA1 on adipogenic differentiation of myotubes as a consequence of FA1-induced down-regulation of the above indicated adipogenic markers. As shown in Figure 3, the addition of soluble FA1 to the myotube cultures inhibited their adipocyte differentiation as assessed by significant reduction in Oil red O staining for fatty acid accumulation (Figure 3A) and marked down-regulation of the expression of adipogenic markers *PPARg2*, *LPL*, and *aP2* (Figure 3B). These data showed that Dlk1/FA1 exerts an anti-adipogenic effect on human myotubes.

### FA1 did not change the effect of PA on the intermediary metabolism

**Figure 4:**
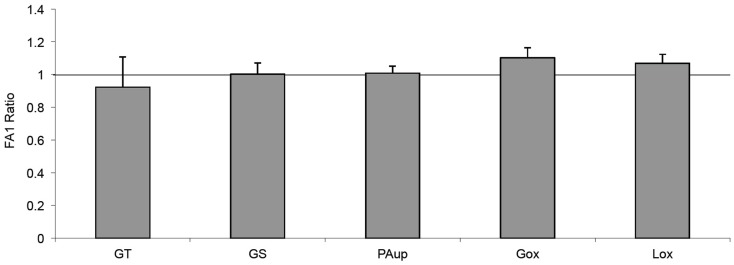
**Effect of FA1 on insulin sensitivity of cultured myotubes exposed to PA**. Cultured myotubes established from lean subjects were differentiated for 4 days under physiological conditions and subsequently exposed to 0.4 mmol/l PA with or without 3.0 μg/ml FA1 for further 4 days, as described in Section “Materials and Methods.” Glucose uptake (GT), glucose oxidation (Gox), glycogen synthesis (GS), lipid uptake (PAup), and lipid oxidation (Lox) were determined at baseline and during acute insulin stimulation. Data represented as ratio of (insulin stimulation/basal) with/without FA1. Data are shown as mean ± SEM. *N* = 8.

In order to clarify whether the inhibitory effect of FA1 on adipogenesis in myotubes could improve their sensitivity for PA-induced insulin resistance, we treated the established cultured myotubes from lean subjects with PA in the presence or the absence of FA1 for 4 days. The incubation of lean myotubes with PA with/without FA1 for 4 days did not show any significant changes in insulin sensitivity parameters for glucose uptake, oxidation, glycogen synthesis, PA uptake, and oxidation (Figure 4).

### Chronic FA1 did not reduce/impair the incorporation of PA into TAG in human myotubes

**Figure 5:**
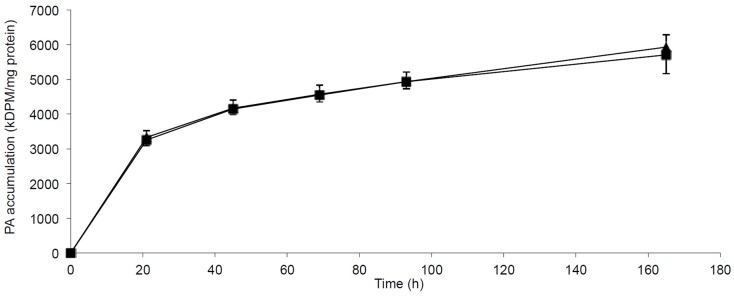
**Accumulation of PA in myotubes induced with/without FA1**. Cultured myotubes from lean subjects were exposed to 0.4 mmol/l PA with/without 3.0 μg/ml FA1 for 7 days and the intracellular accumulation of PA was regularly monitored. The figure illustrates the time course of PA incorporation into myotubes in the presence (◼) and the absence (▲) of FA1. Data are shown as mean ± SEM. *N* = 6.

The incorporation and accumulation of fatty acid in lean myotubes were studied by SPA methodology allowing us to measure the continuous PA incorporation into myotubes at 0.4 mmol/l PA with or without FA1 for 7 days. The accumulation of PA was independent of the presence of FA1 (Figure 5).

## Discussion

We have studied the *in vitro* effect of circulating FA1 on the insulin sensitivity of human myotubes established from lean, obese, and T2D subjects. Our metabolic studies clearly showed that FA1 does not exert a direct effect on insulin sensitivity of glucose and lipid metabolism in myotubes. Instead, chronic exposure of FA1 stimulates the production of pro-inflammatory cytokines by myotubes in association with inhibiting their adipogenic markers.

Cultured human myotubes are the most similar cell system to intact skeletal muscle that can be modulated *ex vivo*. Compared to rodent models, our cultured myotubes expresses the right genetic background as well as the specific skeletal muscle phenotype. The extracellular environment can be controlled precisely and kept relatively constant over time, without interference from systemic homeostatic compensatory mechanisms. We and others have reported several potential intrinsic deficiencies in myotubes from individuals with T2D, i.e., reduced capacity for complete oxidation of labeled PA to CO_2_ compared to control myotubes or reduced insulin mediated increase in glycogen synthesis, and these dysfunctions was reproduced in our present study (Gaster and Beck-Nielsen, 2004; Gaster et al., 2004; Gaster, 2009a).

We and others have shown that the increased serum levels of FA1 was associated with human pathological conditions including neurofibromatosis (5 times higher than the normal range) (Jensen et al., 1999), renal failure (10 times higher) (Jensen et al., 1997), small cell lung cancer patients (10–1000 times higher) (Harken et al., 1999), and estrogen deficiency-induced osteoporosis in post-menopausal women (Abdallah et al., 2011a).

With regard to IR, increasing serum levels of FA1 in mice was shown to be associated with IR through an indirect mechanism involving lipodystrophy-induced IR in peripheral fat (Villena et al., 2008). In accordance, our finding that both insulin sensitivity and lipid metabolism were not *per se* affected by chronic FA1 exposure in human myotubes suggests that myotube is not the primary targeted tissue by FA1-induced IR.

Our data showed that Dlk1/FA1 inhibits the adipocyte differentiation of cultured human myotubes via a mechanism involved the down-regulation of the adipogenic regulatory genes. This finding is consistent with the established inhibitory effect of Dlk1/FA1 on the adipocyte differentiation of 3T3-L1 and mesenchymal stem cells as well as on reducing fat mass *in vivo* where, the increased serum levels of FA1 showed to be negatively correlated with fat mass in mice (Lee et al., 2003; Abdallah et al., 2007b). Moreover, in other species including sheep and pigs, the high circulating level of FA1 due to genetic mutation of *dlk1* region was shown to be associated with reducing fat mass (Freking et al., 2002; Kim et al., 2004).

Several studies suggested a strong link between inflammation and the development of insulin resistance. Activation of inflammatory pathways in fat and skeletal muscles showed to induce local and systemic insulin resistance (Cai et al., 2005; Plomgaard et al., 2005; Bastard et al., 2006). In this study, the stimulatory effect of Dlk1/FA1 on increasing the production of IL-6 and monocyte chemotactic protein (MCP)-1 by myotubes did not seem to be involved in pathogenesis of insulin resistance in T2D patients (Kalupahana et al., 2012) due to the fact that we could not detect any differences in the FA1-induced pro-inflammatory cytokines in myotubes between T2D and lean and obese controls. Thus, our data could not identify a link between the FA1-induced inflammation in myotubes and insulin resistance. On the other hand, we have recently identified the Dlk1/FA1-induced inflammation as a potential mechanism that mediates the inhibitory effect of Dlk1/FA1 on osteoblast and adipocyte differentiation of human mesenchymal stem cells (Abdallah et al., 2007a) as well as its stimulatory effect on osteoclast differentiation from hematopoietic stem cells in bone marrow (Abdallah et al., 2011b). Thus, it is plausible that the anti-adipogenic effect of Dlk1/FA1 on myotubes is mediated though a mechanism involved increasing the production of cytokines by Dlk1/FA1.

The association between Dlk1/FA1 expression and inflammation has also been demonstrated in a recent report that demonstrated the stimulatory effect of Dlk1/FA1 protein on pro-inflammatory cytokines by immune and fat cells (Chacon et al., 2008). Furthermore, several studies support the involvement of Dlk1/FA1 in general biological processes associated with stimulating inflammatory responses including detection of high Dlk1/FA1 expression levels during ear wound healing (Samulewicz et al., 2002), during liver regeneration in liver injured mice (Jensen et al., 2004), and by satellite cells during muscle regeneration (Crameri et al., 2004). In conclusion, FA1 *per se* seems to not induce insulin resistance or worsen pre-existing insulin resistance in human myotubes, but shows its effect at the gene level by down-regulating the expression of adipogenic differentiation markers and up-regulating pro-inflammatory cytokines.

## Conflict of Interest Statement

The authors declare that the research was conducted in the absence of any commercial or financial relationships that could be construed as a potential conflict of interest.

## Abbreviations

FFA, free fatty acids.
